# Case of Massive Hydatid Pulmonary Embolism Incidentally Discovered in a 56-Year-Old Woman with Posttraumatic Abdominal Pain

**DOI:** 10.1155/2018/7831910

**Published:** 2018-05-13

**Authors:** Zied Mezgar, Mariem Khrouf, Houda Ben Soltane, Mohamed Mahjoub, Sihem Ben Fredj, Amel Amara, Maher Jedidi, Samar Hajer Sandid, Sarra Zaouali, Ines Waz, Asma Saada, Mehdi Methamem

**Affiliations:** ^1^Department of Emergency Medicine, University Hospital Farhat Hached, Sousse, Tunisia; ^2^Department of Hospital Hygiene, University Hospital Farhat Hached, Sousse, Tunisia; ^3^Department of Epidemiology, University Hospital Farhat Hached, Sousse, Tunisia; ^4^Department of Epidemiology, University Hospital Fattouma Bourguiba, Monastir, Tunisia; ^5^Department of Legal medicine, University Hospital Farhat Hached, Sousse, Tunisia

## Abstract

Hydatid pulmonary embolism rarely occurs. It arises from the rupture of a hydatid heart cyst or the opening of a visceral hydatid cyst into the venous circulation. We report a case with pulmonary hydatidosis resulting in a massive bilateral pulmonary embolism in a 56-year-old woman with history of hepatic echinococcosis. A brief overview of clinical features and radiologic findings is presented.

## 1. Introduction

Hydatid disease is frequently encountered in the sheep and cattle-raising regions of the world, being most common in Australia, New Zealand, South Africa, South America, and the Mediterranean countries of Europe, Asia, and Africa [1]. Echinococcosis is endemic to Tunisia and a very important disease. Human infection with* E. granulosus* leads to the development of one or more hydatid cysts located most often in the liver and lungs. However, pulmonary artery embolism due to hydatid cyst is an extremely rare entity that can be seen secondary to cardiovascular system invasion. It is usually seen in cardiac involvement but it can be also seen due to inferior vena cava (IVC) or hepatic vein invasion [2].

## 2. Case Report

A 56-year-old woman presented to our emergency department with a posttraumatic abdominal pain as she was punched and hit with a rock several times on both the right upper quadrant of her abdomen and the lower part of her chest 2 hours prior to her admission to our department. She was otherwise well with a medical history of a surgical excision of a hepatic hydatid cyst at the age of 40.

There was no history of recurrent cough, hemoptysis, dyspnea, or chest pain.

The temperature was 37, the pulse was 88, the respiratory rate was 20, the oxygen saturation was 99% while the patient was breathing the ambient air, and the blood pressure was 14/9 mmHg.

On examination, the patient appeared well. No signs of rib fracture were found. On auscultation the lungs were clear. No abdominal nor chest bruises were found. Liver and spleen were palpable with tenderness at the right hypochondriac region of the abdomen.

The laboratory investigations were all within normal limits. Arterial-blood partial pressures of oxygen (PaO2) and carbon dioxide (PaCO2) were 68.9 and 36 mmHg, respectively.

The electrocardiogram showed sinus tachycardia with no axis deviation nor T wave or ST segment abnormalities.

**Figure 1:**
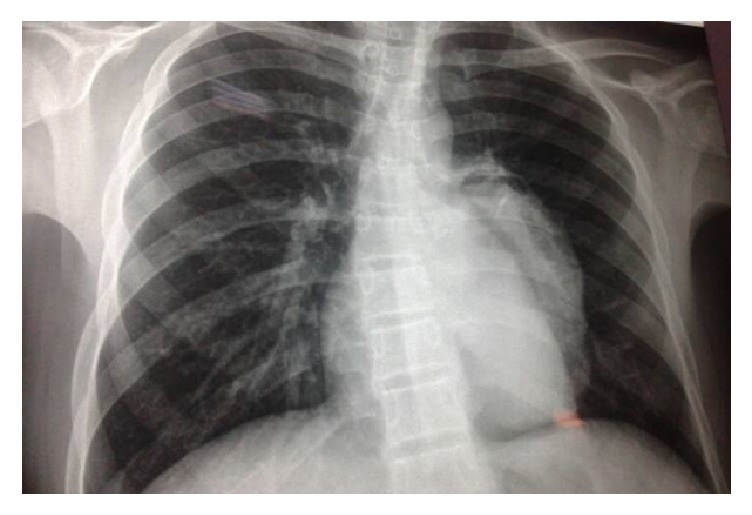
Front view chest X-ray showing a rounded prominence of the left cardiac border with the presence of a radiolucent rim at its superior aspect (crescent sign).

**Figure 2:**
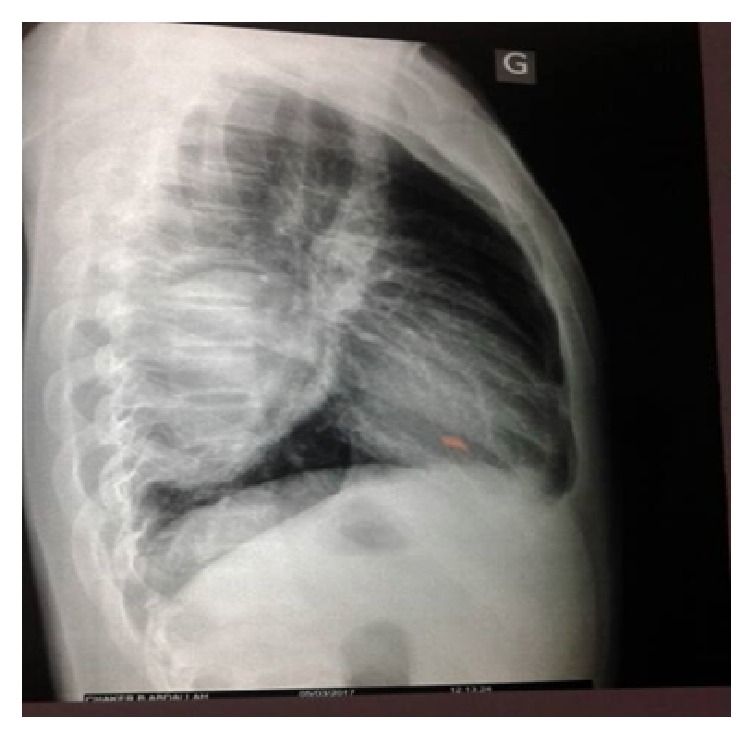
Lateral view chest X-ray showing a well-defined rounded opacity was in the retrocardiac space.

In the chest X-ray, there was a rounded prominence of the left cardiac border with the presence of a radiolucent rim at its superior aspect (crescent sign) (Figure 1). In the lateral view, well-defined rounded opacity was in the retrocardiac space (Figure 2).

**Figure 3:**
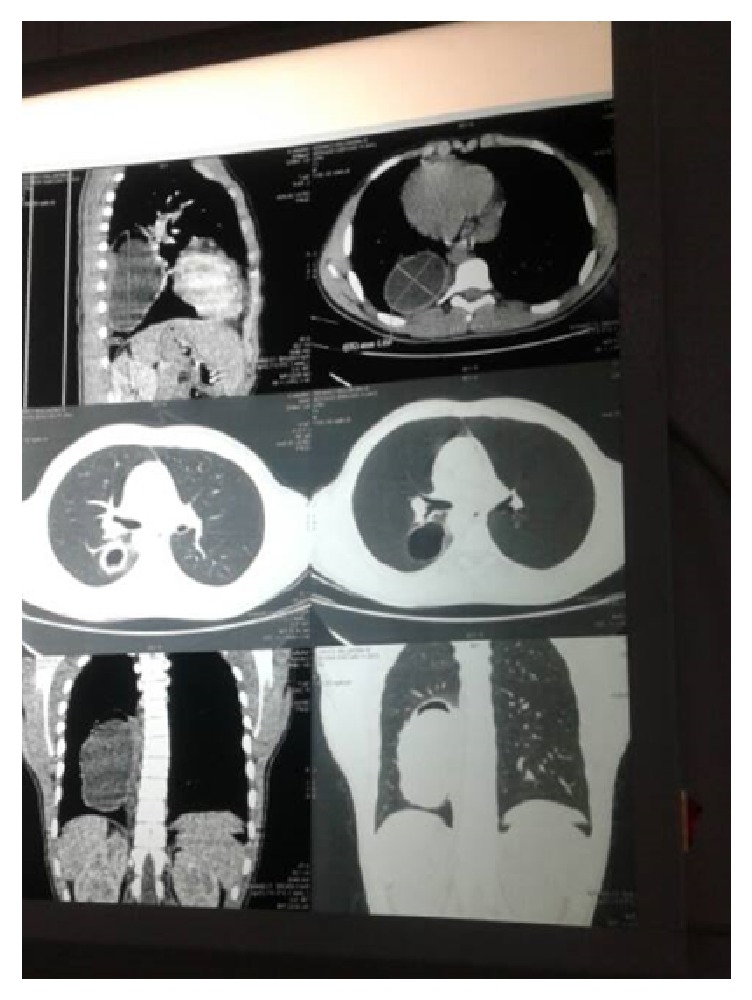
Chest CT scan with a well-circumscribed fluid attenuation lesion with homogenous content and smooth, hyperdense walls located in the lower lobe of the right lung measuring 30 by 20 by 27 cm.

**Figure 4:**
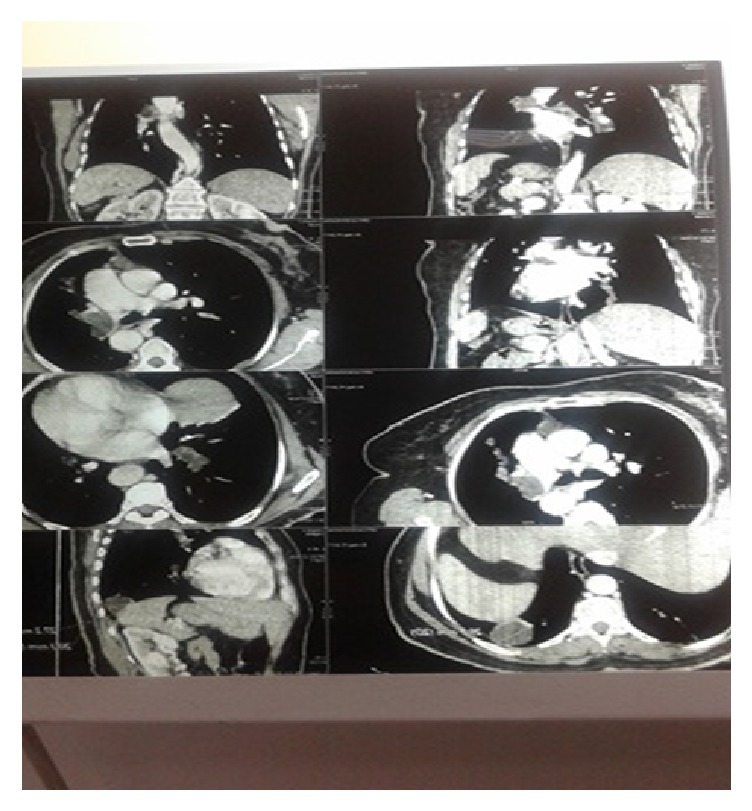
CT chest scan with a 31 cm dilation of the pulmonary trunk with a partial occlusion of the left pulmonary artery and a complete occlusion of the right pulmonary artery by a fluid attenuation lesion and a subpleural cyst of the inferior lungular segment measuring 19 mm.

CT scan of the chest revealed (Figure 3) the following:

(i) 31 cm dilation of the pulmonary trunk with a partial occlusion of the left pulmonary artery and a complete occlusion of the right pulmonary artery by a fluid attenuation lesion (Figure 4);

(ii) a well-circumscribed fluid attenuation lesion with homogenous content and smooth, hyperdense walls located in the lower lobe of the right lung measuring 30 by 20 by 27 cm (Figure 4);

(iii) a subpleural cyst of the inferior lungular segment measuring 19 mm (Figure 4).

Abdominal ultrasonography and CT scan were normal.

The radiologic investigations concluded massive pulmonary hydatid embolization with pulmonary echinococcosis.

A transthoracic echography showed no signs of cardiac cysts and there were no signs of pulmonary hypertension.

The patient has been admitted to the Department of Cardiology in University Hospital Farhat Hached for treating the hydatid pulmonary embolism. The cardiac surgeons decided to abstain from a surgical intervention.

## 3. Discussion

Echinococcosis or hydatid disease is caused by larvae of the tapeworm Echinococcus. In cystic echinococcosis, humans constitute an accidental host and are usually infected by handling infected dogs [3]. The sites of hydatid cyst formation within human tissues are liver 60–75%, lung 15–25%, and the remaining parts of the body such as bone, brain, and mediastinum 10–15% [4].

The hydatid cysts that form as a direct consequence of the initial infestation are considered primary cysts. If a primary cyst ruptures and results in new hydatid cysts forming, this is referred to as secondary hydatidosis, which may be diagnosed incidentally in an asymptomatic patient or present secondary to a complication of the hydatid cyst [4].

In our case, the investigations of a posttraumatic abdominal pain led not only to the discovery of pulmonary echinococcosis but also to massive bilateral hydatid pulmonary embolization.

Hydatid pulmonary embolism can occur as a result of hepatic or abdominal cyst rupturing into the hepatic veins or the inferior vena cava or directly from the ruptured cyst in the right cardiac chambers. Surgical and autopsy findings indicate that the embolism is caused by vesicles or daughter cysts that act purely mechanically by obstructing the blood flow [5].

The pulmonary embolism detected in our case may probably have resulted, on the one hand, from the accidental injury to the case's inferior vena cava during earlier liver surgery which may have allowed the spread of daughter cysts within systemic circulation. On the other hand, according to the CT pictures the cyst would be complicated and there was most likely direct rupture of the hydatid cyst into the left pulmonary artery.

Hydatid pulmonary embolism has been classified according to three types of outcome: (i) acute fatal embolism; (ii) subacute embolism and pulmonary hypertension and death in less than a year; and (iii) chronic pulmonary hypertension [4]. According to the transthoracic echography, the case reported here differs from those previously described lacking any signs of pulmonary hypertension, and it seems possible that their pulmonary perfusion was maintained via the bronchial arteries [6]. Nevertheless, the transthoracic echography is an instantaneous medical imaging, which depends on the doctor's skills and experience.

The thoracic imaging may have an important income to guide the diagnosis, especially the helical CT, angiography, and MRI. They can locate the hydatid cysts, study their morphologies, and show their extension to the vascular branches. They may indicate endoluminal defects at the level of the pulmonary artery and/or its branches. On CT scan, the complicated intra-arterial embolism appears as a fluid mass density with limited enhancement of the wall after contrast injection. This was the case in our patient [7].

## 4. Conclusion

In summary, pulmonary embolism caused by* E. granulosus* should be considered in patients who have undergone surgery for hepatic or cardiac cyst removal, especially in regions where echinococcosis is endemic [8].

The case reported is an example of a hydatid hepatic cyst, resulting in a secondary (pulmonary) hydatidosis and massive bilateral hydatid embolization incidentally discovered after an abdominal minor trauma, by means of computed tomographic chest and abdominal imaging. Consideration of this uncommon manifestation of hydatid disease will allow for its diagnosis and treatment and may avert life-threatening recurrent emboli.
